# Draft Genome Sequence of *Escherichia coli* Strain SEC470, Isolated from a Piglet Experiencing Diarrhea

**DOI:** 10.1128/genomeA.00088-16

**Published:** 2016-03-10

**Authors:** Hongbin Liu, Li Song, Yinghua Cai, Yashan Wang, Lei Yu

**Affiliations:** a China Animal Disease Control Center, Beijing, China; b China Institute of Veterinary Drug Control, Beijing, China

## Abstract

*Escherichia coli* strain SEC470 is a diarrhea-causing strain, isolated from a piglet experiencing serious diarrhea in Jingxi Province, China. Here, we present the draft genome of this strain, which provides the genetic basis for exploring the mechanism of enterotoxigenic *E. coli* infections.

## GENOME ANNOUNCEMENT

Enterotoxigenic *Escherichia coli* (ETEC) is the most common cause for travelers’ disease and a major cause of infectious diarrhea for infants and newborn animals in developing countries (1). Here, we report the newly sequenced *E. coli* strain SEC470, isolated from a piglet experiencing serious diarrhea in Jingxi Province, China (2, 3).

*E. coli* SEC 470 is an O4 serotype strain of ETEC that causes diarrhea in piglets (4). *E. coli* SEC 470 genomic DNA was extracted using a QIAamp DNA mini kit (Qiagen) according to the manufacturer’s protocol. About 3 ng of genomic DNA was used to generate a library using the Nextera XT kit (Illumina). Micro-Seq Enterprises sequenced the libraries on a MiSeq sequencing system (Illumina). The sequencing generated approximately 6 million read pairs, constituting about 100-fold coverage of the genome. A *de novo* assembly (SOAPdenovo version 2.04) was applied to assemble the raw FASTQ sequences into a total length of 5.15 Mb (about100-fold coverage) (5). Annotation was done using the Rapid Annotations Using Subsystems Technology (RAST) server (6).

The genome sequence of *E. coli* SEC 470 comprises 5,153,435 bp, and the annotation revealed 5,192 open reading frames. The availability of the *E. coli* SEC 470 draft genome sequence provides opportunities for exploring the mechanism of ETEC infections.

### Nucleotide sequence accession number.

This whole-genome shotgun project has been deposited at GenBank under the accession number CP013962. The strain is available from Lei Yu (China Animal Disease Control Center, Beijing, China).
